# Editorial Chit-Chat

**Published:** 1872-04

**Authors:** 


### EDITORIAL CHIT-CHAT.

CONKEY, the Sculptor, is spending the winter at Ralston, Pa., where he is engaged in modeling several busts for parties in Chicago.

PERSONAL.—Our former townsman, O. W. Palmer, Esq., is to become the Secretary of the Manhattan Fire Insurance Co., of New York. Mr. Palmer is regarded as one of the most efficient insurance men in the city.

DON'T VOTE RIGHT.—One would suppose that the newly elected Alderman from the Fifth Ward, belonged to "the other side of the river," judging from the manner in which he voted on the bridge question, in the common council, recently.

BACK NUMBERS.—We have a few, complete volumes of the Bistoury from 1869. Have also several handsomely bound copies, from the date it was first issued in magazine form, until the present time, which we can furnish for $3.00 a copy, bound, or $1.50 for the set unbound.

EXQUISITE.—One of the finest specimens of art ever exhibited in this city, can now be seen in Roosa's art window, on Lake St. The piece represents a Priest blessing the Earl of Stafford prior to his execution, and is the work of Miss C. Sinnett of our city, an artist of rare culture and genius. We also saw a portrait of a little child, in Mr. Hart's window on Water St., painted by the same artist, which will at once convince the observer that Miss Sinnett is an artist of no ordinary merit. We have seen very few better pictures, and many inferior ones, the efforts of the best artists of New York City.

NOVEL FISHING.—An old chap, off the coast of Norway, kills whales by means of an explosive harpoon. The harpoon is loaded with a pound or more of powder, is fired at the whale from a small cannon, and the moment it penetrates the poor whale, explodes, which either frightens him to death, or blows him out of water—one or both. We do not recommend this plan for trout fishing, and hope none of our piscatorial friends will try any such confounded nonsense, this summer,—not if we have to accompany them.

---

SUPPLY OF MEDICAL MEN.—A foreign medical journal informs us that there is one physician for every 5,532 inhabitants of England and Wales. In this country, according to the last census, there is a doctor for every 527 of the people. It strikes us that this is a little over doing the healing art in America, and that some of our half-starving brethren had better migrate to the countries first named, where there seems to be abundant room for those who lack the requisite number of patients to make practice remunerative.

---

UNIQUE DEVICE.—A Missouri genius, who doubtless migrated from Connecticut, has invented a new means for automatically closing his bee-hives at night, so as to exclude the bee-moth, which only attacks the bees at night. And this is the way he accomplishes it: "The invention consists in so combining and arranging a poultry roost with the gates of one or more bee hives, that the perching of the poultry upon the roost will serve to close the hive." In this arrangement it is necessary for the "busy bee" to be in on time, or the hens, who always keep good hours, may have the front door locked, and so exclude them for the night.

---

ERRORS OF EXPERTS.—The fact that "doctors disagree," is an old, but none the less truthful proverb, and one that would apply equally well to chemists. Were we compelled to read the report of another trial similar to Mrs. Wharton's, in which many of the most noted chemists of the land were arrayed, one against the other, one party asserting that poison *was* found in General Ketchum's stomach, and that the poison was tartarized antimony, backing up their assertions by satisfactory chemical proofs and experiments in the presence of the jury; while the opposing experts, with equal confidence and as startling experiments, declared no poison to be present, and that death ensued from natural causes; we confess we should be a trifle fuddled as to whether chemistry was really a science or not. It is quite probable that the jury was sorely puzzled, if not completely disgusted, with the opinions of these scientific experts, and proves that men of supposed scholarly attainments, and who have been thrust into high places, do not remain there by virtue of their fitness for such positions.

---

"FRASIER HOUSE."—The American Hotel, near the depot, is being thoroughly overhauled—repapered, repainted, and rebuilded in many parts. It is also to be newly furnished throughout, and opened about the 15th of April, under a new name and new management. It will be known in future as the "Frasier House," with Mr. F. A. Frasier as landlord, and Mr. Chester Spaulding as general manager. Both these gentlemen have hosts of friends on the line of the Erie, and intend to keep a first class house, with the best table and beds to be found in any hotel in the State.

---

TOO PLEASANT, BY FAR.—The Agricultural editor of the *Advertiser* informs us this morning, (March 15th), that trout fishing may be indulged in to-day, without violation of law. Taking an observation of the thermometer, we find it nearly at zero,—the ground is covered with three inches of snow,—wind blowing like fury, and twenty-four inches of ice on the river, while many of the creeks are frozen to the bottom. In view of these facts, how delightful it would be to be seated upon the mossy bank of some babbling brook, luring the wary trout from his hiding place, by gracefully gliding the deceptive fly over a foot or so of ice. We are as enthusiastic a fisherman as any we wot of, but excuse us this morning, Sam.

---

LAWS RELATING TO NEWSPAPERS.—We have been asked to give the law, as it stands, relating to newspapers and subscribers:

1. Subscribers who do not give express notice to the contrary, are considered wishing to continue their subscription.

2. If subscribers order the discontinuance of their periodicals, the publishers may continue to send them until arrearages are paid.

3. If subscribers neglect or refuse to take their periodicals from the office to which they are directed, they are held responsible till they have settled their bill, and ordered them discontinued.

4. If subscribers move to other places without informing the publishers, and the papers are sent to the former places, they are held responsible.

5. The courts have decided that refusing to take periodicals from the office, or removing and leaving them uncalled for, is *prima facie* evidence of intentional fraud.

6. Any person who receives a newspaper and makes use of it whether he has ordered it or not, is held in law to be a subscriber.

---

COWS.—For some time we have been searching for a good cow; not a Durham, Alderny, or any other fancy breed, with symmetrical bodies, short horns, and little milk; but the common, old-fashioned sort; those to the manor born, homely mottled, scrawny; with long, crooked, or crumply horn, like unto the one that tossed the dog that worried the historical feline; in short, the only cow good for milking purposes, a common, native cow.—Well, our wants having been made known, we were astonished to find how many good citizens had remarkable cows to sell,—cows that would give enormous and immoderate quantities of milk. We were amazed to find so many that could give easily, and with little food, from 18 to 24 quarts of rich milk per diem. This was the *only* sort that were offered us, and not wishing to engage in the dairy business, were compelled to decline buying a cow at all.—and at this writing are purchasing the peddled, chalk-and-water-sort-of-stuff, as of yore. Speaking of cows, reminds us that they have a tree in Venezuela, which is called the "cow tree," and which, when the trunk is bored, furnishes a bland and nourishing milk, from the surface of which a rich cream is collected, after having stood for the usual time. What a pity that our northern climate is not congenial to this lactiferous tree, so that every man could have his own cow,—"a free, easy milker,"—standing in his garden, or ornamenting his front yard, with no nuisance of a boy about to drive said cow to a distant and miserable pasture! O, for an acclimatization society, to furnish us with the hornless and legless South American cow!

THE RIVER NUISANCE.—Now that we have a new board of health, it is to be hoped that some means will be devised by them, to prevent further contagion from the constantly accumulating filth in the river, between the railroad and Lake street bridges. The buildings on the bank of the river, between these two points, are densely inhabited by families, who convert the river into a sort of swill-tub, for the reception of all manner of filth and nastiness. The grocers deposit their decayed vegetable matter here, while persons all along both sides of Water street, are in the habit of using the river as a general receptacle for offal of all descriptions. To add to the intolerable effluvia emanating from this mass of filth, the privies used by these various families, are built so as to project over the river. When these edifices receive the brilliant rays of a July sun, the perfume (?) emanating from them, is of a character that compels the closing of all windows and doors, having an outlook in that direction.

There exists not the least doubt in our mind, that many of the fatal cases of dysentery which occurred among shop-keepers and the families inhabiting the river buildings, last summer, were the result of contagion arising from decomposed vegetable matter, deposited in the river.

We hope the board of health will take immediate action in this matter, and cause the accumulated filth already deposited, to be removed, the river dredged under these privies, and prohibit the further deposit of the refuse of the family, in this too convenient channel. Let there be a city scavenger appointed, whose duty it shall be to cart off daily, the garbage, which the family should deposit in barrels, provided for the purpose. Cause the property holders to build proper water closets, to be connected with the street sewer, and remove the present nasty buildings, used for that purpose. If something of the sort is not done, and that right speedily, we predict that our city will be visited by miasmatic fevers and dysenterys, this summer, that will *compel* our city authorities to take some action in this matter, perhaps after many valuable lives have been sacrificed.

---

LEFT OUT.—Considerable editorial matter has been crowded out of this number. We had prepared notices of our exchanges, which must lie over with other matter, until next issue.

